# Economic Crisis and Sexually Transmitted Infections: A Comparison Between Native and Immigrant Populations in a Specialised Centre in Granada, Spain

**DOI:** 10.3390/ijerph17072480

**Published:** 2020-04-05

**Authors:** María Ángeles Pérez-Morente, Adelina Martín-Salvador, María Gázquez-López, Pedro Femia-Marzo, María Dolores Pozo-Cano, César Hueso-Montoro, Encarnación Martínez-García

**Affiliations:** 1 Faculty of Health Sciences, University of Jaén, 23071 Jaén, Spain; mmorente@ujaen.es; 2 Faculty of Health Sciences, University of Granada, 52005 Melilla, Spain; 3 Faculty of Health Sciences, University of Granada, 51001 Ceuta, Spain; mgazquez@ugr.es; 4 Faculty of Health Sciences, University of Granada, 18016 Granada, Spain; pfemia@ugr.es (P.F.-M.); pozocano@ugr.es (M.D.P.-C.); emartinez@ugr.es (E.M.-G.)

**Keywords:** sexually transmitted infections, economic recession, transients and migrants

## Abstract

This study aimed to analyse the influence of the economic crisis on the prevalence of sexually transmitted infections (STIs) in the immigrant population compared to the native population. A cross-sectional study was conducted by reviewing 441 clinical records (329 Spanish nationals and 112 non-Spanish nationals) of individuals who, between 2000 and 2014, visited an STI clinic in Granada and tested positive for an infection. Descriptive statistical analyses were performed, and infection rates, odds ratios, and 95% confidence intervals (CIs) were calculated. The mean age was 28.06 years (SD = 8.30; range = 16–70). During the period 2000–2014, the risk of being diagnosed with an STI was higher among non-Spanish nationals than among Spanish nationals (odds ratio (OR) = 5.33; 95% CI = 4.78–6.60). Differences between both populations were less marked during the crisis period (2008–2014: OR = 2.73; 95% CI = 2.32–3.73) than during the non-crisis period (2000–2007: OR = 12.02; 95% CI = 10.33–16.17). This may be due to underreporting of diagnoses in the immigrant population. Immigrants visiting the STI clinic in Granada are especially vulnerable to positive STI diagnoses compared to the native population.

## 1. Introduction

Sexually transmitted infections (STIs) are a global public health problem. According to the World Health Organization (WHO), more than one million people contract an STI every day. An estimated 357 million people become infected with chlamydia, gonorrhoea, syphilis, or trichomoniasis each year [1].

According to the latest epidemiological report from the European Centre for Disease Prevention and Control, the number of gonorrhoea infections had increased by 17% in 2017 compared to the previous year. The United Kingdom was the country with the highest proportion of confirmed cases (75 per 100,000 inhabitants), followed by Ireland (47), Denmark (33), Iceland (29), Norway (27), and Sweden (25). The countries with the lowest proportion of reported cases (<1 per 100,000) were Bulgaria, Croatia, Cyprus, Poland, and Romania. There were 33,189 (7.1 cases per 100,000 inhabitants) new cases of syphilis. The highest rate was found in Iceland (15.4 cases per 100,000 inhabitants), followed by Malta (13.5), the United Kingdom (11.8), and Spain (10.3). The lowest rates (<3 cases per 100,000 inhabitants) were observed in Croatia, Cyprus, Estonia, Italy, Portugal, and Slovenia. Furthermore, 25,353 people were diagnosed with Human Immunodeficiency Virus (HIV) in the European Union (6.2 cases per 100,000 inhabitants) [2].

In 2016, in Spain, the STI with the highest incidence rate was chlamydia trachomatis (17.85/100,000), followed by gonorrhoea (13.8/100,000), and syphilis (7.22/100,000). In the case of syphilis, it has been stabilised since 2011. However, gonococcal infection rates have increased steadily from 2001 to 2016, from 2.02/100,000 to 13.89/100,000 [3].

Men who have sex with men (MSM), sex workers, transgender people, intravenous drug users, and immigrants have been identified as the key populations at highest risk of contracting an STI [4].

Increased migration has contributed to the spread of HIV, Hepatitis B Virus (HBV), and other STIs, with the vast majority of cases occurring in migrants from low- and middle-income countries who have moved to high-income countries. Most interventions in the United States of America, Australia, and Europe focus on individual behaviour rather than on broader sociocultural factors [5].

According to the Spanish National Statistics Institute (INE, by its Spanish acronym), the immigrant population in Spain in 2018 increased by 100,764 to 4,663,726, an increase 23% larger than in 2017 [6]. In 2017, 36.1% of new Human Immunodeficiency Virus (HIV) infections were diagnosed in non-Spanish individuals, being more frequent in the Latin American population (19%) [7]. In a study conducted at a hospital in Madrid, half of the 371 new HIV diagnoses were made in immigrants [8]. The immigrant population constitutes a very diverse and particularly vulnerable group due to the socio-cultural context, the language barrier (in some cases), their economic level, and their employment and legal status in Spain. In addition, the WHO points out that insufficient data on STIs at the local level compromises the global response to this problem [4].

Periods of economic crisis represent one of the factors that increase the proportion of socially and economically vulnerable citizens, including immigrants [9]. Different studies have analysed the effects of the economic crisis on the immigrant population in several European countries [10,11,12,13]. The economic situation in Spain in recent years has increased the impact of communicable diseases, especially on the most vulnerable populations [14,15,16]; thus, hindering healthcare delivery to immigrants [17].

The present research is in line with these studies and aims to analyse STI-related differences between the pre-crisis and crisis periods by comparing the native population with the immigrant population, of those who visited the Sexually Transmitted Disease and Sexual Health Clinic in Granada (Spain). Population rates have been taken into account in this comparison, which we consider to be a differentiating feature of this study, with respect to previous studies.

The objectives of this study were as follows: to describe the evolution of STIs in the non-Spanish population in comparison to the native population living in Granada (Spain); to explore, in the former group, the potentially higher risk of contracting some of these infections in comparison to the rest of the population, using the period of economic crisis as a variable of interest.

## 2. Materials and Methods

An observational study was conducted by analysing the cases of service users diagnosed with STIs who visited the Sexually Transmitted Disease and Sexual Health Clinic in Granada, Spain, between 2000 and 2014, inclusive. This specialised centre, attached to the Andalusian Health Service (SAS, by its Spanish acronym), is the referral service for the entire province of Granada and, according to INE data, during the years analysed, a yearly average total of 550,000 individuals aged between 15 and 64 years old have used their services [18].

These records were taken from a randomly generated database within a larger project from which this study derives. A sample size was calculated to detect differences in the basic variables of STI presence in patients with a new clinical record. This calculation was made in order to detect differences in a binary variable, seeking to detect differences of 20% in two years, with a power of 80% and applying an error of *α* = 5% to the test. The sample size required to detect this difference was 97 clinical records per year. The sample was obtained from a database of new records for each year, from which the first and the last record number of each year were taken using systematic random sampling without replacement.

This database contains the clinical records of 1437 adult users without cognitive impairment whose reason for visit was suspicion of, or confirmed presence of, an STI. The sample analysed in the present study, which is a sub-sample of the aforementioned sample, consists of subjects who had been diagnosed with an STI.

Data were collected from the clinical records using four categories: symptoms; control; contact follow-up; and HIV. The country of birth was the independent variable (Spanish nationals vs. non-Spanish nationals), which was determined by means of an official identification document (i.e., national identity card, residence permit, work permit, or passport). Time was the main explanatory covariate (i.e., the 2000–2014 period), in which 2008 was considered to be the onset of the economic crisis in Spain, as numerous studies indicate [9,10,19]. Other variables included were: (a) socio-demographic variables: sex (male/female), age in years (analysed as a continuous variable), occupation (sex worker/former sex worker/other), employment status (employed/unemployed), level of education (no education or primary education/secondary education/higher education), living with a partner (yes/no), and sexual orientation identity (heterosexual/bisexual/homosexual); (b) variables related to clinical care received: reason for visit (according to the reasons provided in the clinical history), previous treatment (yes/no), number of subsequent visits, number of new subsequent episodes; and (c) risk factors for contracting STIs: regular partner having symptoms (yes/no), period of time since last sexual contact without a condom, number of partners in the last month, number of partners in the last year, lifelong sexual history (number of sexual partners throughout lifetime), drug use (yes/no), previous STIs, and age of first sexual intercourse in years (analysed as a continuous variable). The following variables, registered in the clinical records as nominal variables were transformed into quantitative variables for ease of analysis: period of time since last sexual contact without a condom, number of partners in the last month, number of partners in the last year, and lifelong sexual history.

To analyse the effect of the financial crisis on STI diagnoses, the annual rates of STI diagnosis per 100,000 inhabitants were calculated for Spanish nationals and non-Spanish nationals using the direct method, taking as the denominator the number of individuals over 15 years old residing in the province of Granada for each group, according to the data published by the INE in the continuous annual census [18]. These rates were plotted to highlight trends. Odds ratios (ORs) of STI diagnoses between non-Spanish nationals and Spanish nationals and 95% confidence intervals (CIs) were calculated for each year of the pre-crisis (2000–2007) and crisis (2008–2014) periods, as well as for the total study period (2000–2014).

Statistical analyses were conducted using the Statistical Package for the Social Sciences 22.0 (SPSS; International Business Machines Corporation [IBM], 2016, Armonk, NY, USA).

The study protocol was approved by the Biomedical Research Ethics Committee of the province of Granada (research protocol approved on 12 February, 2012 and 1 April, 2015), as well as by the Management Directorate of the Granada-Metropolitano Health District, to which the centre where data were collected is attached.

## 3. Results

The total number of service users diagnosed with an STI was 441, of whom 329 were Spanish (74.6%) and 112 (25.3%) were immigrants. The mean age was 28.06 years (SD = 8.30; range = 16–70). Table 1 shows the characteristics of the sample separated by nationality (Spanish vs non-Spanish).

Table 2 shows the main diagnoses identified by population group, the most frequent being Human Papilloma Virus (HPV) infection. Non-Spanish nationals were disproportionately diagnosed with Gardnerella (16.1%) and syphilis (5.4%), whereas Spanish nationals were disproportionately diagnosed with Molluscum contagiosum (10.6%) and gonococcal infection (6.7%). The proportions observed in the case of HIV infection are similar in both groups. However, there is a greater proportion of Herpes simplex virus infection in Spanish nationals and a greater proportion of HBV infection in non-Spanish nationals.

**Figure 1:**
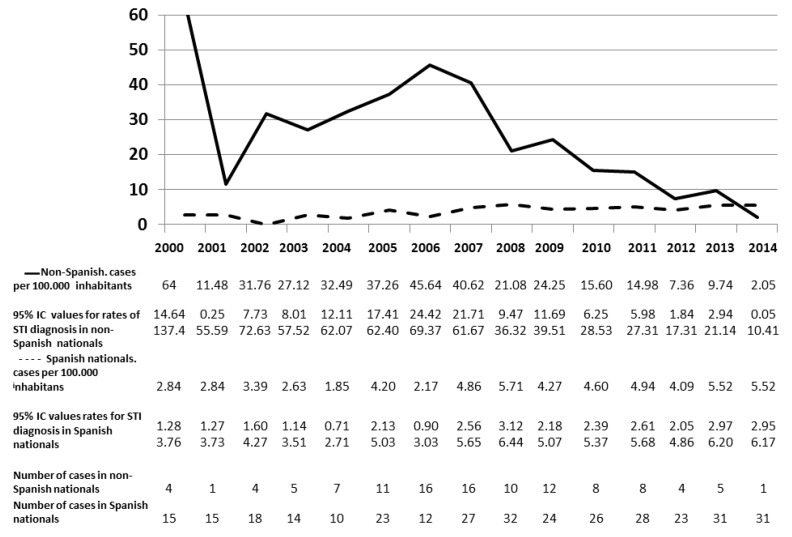
Annual distribution of STI rates for Spanish nationals and non-Spanish nationals aged between 15 and 64 years old. Granada, 2000–2014. STIs: sexually transmitted infections. CI: confidence interval.

The rates of STI diagnoses among the non-Spanish population residing in the province of Granada were higher than among the Spanish population in all the years analysed, with the exception of 2014 (2.05 vs. 5.52) (Figure 1).

In both the pre-crisis (2000–2007) and crisis (2008–2014) periods, as analysed separately, as well as for the entire time period analysed as one, there was a higher risk of being diagnosed with an STI among immigrants than among Spanish nationals. However, the difference observed between the two populations was less pronounced during the crisis period (Table 3).

## 4. Discussion

Regarding the socio-demographic profile of the sample analysed, it is worth noting that, in the immigrant population, a greater proportion of patients were women compared to in the Spanish population. However, the mean age of the sample was very similar in both population groups, being between 27 and 28 years old. There was a higher proportion of individuals with higher education in the native population than in the immigrant population. The high percentage of sex workers or former sex workers in the immigrant population analysed stands out. In reference to the number of partners in the previous month, in the previous year, and throughout their sexually active lives, the mean values of all three of these variables are higher in immigrants than in Spanish nationals.

The most frequently diagnosed STI, in both immigrant and native populations, was HPV infection. This is consistent with a study on STIs conducted in the same region, Andalusia [20], in which HPV infection was the most frequent infection as early as 2009. With respect to other diagnoses, such as hepatitis B and syphilis, also reported in other studies, the results are consistent with another study [21] that reports a higher prevalence of hepatitis B in the immigrant population compared to the native population. In contrast, the pattern is different for syphilis, with a higher prevalence in the native population than in the immigrant population.

Based on the results obtained from the analysis of the crisis and pre-crisis periods, it can be observed that the risk of being infected with an STI is greater in the non-Spanish population throughout the entire period analysed. In addition, the proportion of patients who were not Spanish nationals experienced a linear upward trend until 2012. In this year, the trend seemed to reverse, leading to a progressive reduction of the proportion of patients who were not Spanish nationals, compared to what it had been prior. As a result, the described profile points to a reducing effect on the risk for non-Spanish nationals relative to Spanish nationals during the crisis years. This trend can also be observed in the chart comparing the changes in STI rates. In the Spanish population, the rate of STIs remains relatively stable throughout the period studied. However, the rate of STIs in the non-Spanish population is higher and more erratic in the pre-crisis period than in the crisis period, during which this rate seems to stabilise at a level closer to that of the Spanish population.

There may be several explanations for the apparent drop in risk of STI diagnosis during the crisis period compared to the pre-crisis period, which are consistent with a potential underestimation of STI diagnosis in the immigrant population during the crisis years. For instance, the deteriorating social and working conditions following the onset of the crisis, which fundamentally affected the poorest and most vulnerable areas of society, including immigrants [22,23], may have caused many immigrants in Spain to return to their countries of origin [24] and slowed down the arrival of immigrants in Spain [25]. A previous study concluded that, between 2006 and 2012, the health status of the immigrants who had arrived in Spain prior to 2006 was worse in comparison to that of the native population. A possible explanation for this may be the loss of the healthy immigrant effect during the most severe impact of the economic crisis on immigrants [26]. In addition, the passage in 2012 of the Royal Decree-Law 16/2012, on urgent measures to guarantee the sustainability of the Spanish National Health Service and improve the quality and safety of its services [27], restricted access to health care for illegal immigrants. The fear of being diagnosed with an STI and its potential ramifications (e.g., losing their job and/or residence permit) stands in the way of carrying out diagnostic tests in the immigrant population, especially when access to treatment and healthcare is being restricted.

The effect of austerity policies on the reduction of preventive strategies should also be taken into account. The decrease in use of contraceptives since 2007, the absence of protective measures against STIs in one fifth of occasional or sporadic relationships, as well as the increasing incidence of syphilis, gonorrhoea, and HIV in certain groups of individuals, demonstrate that there is a need to place more emphasis on preventive strategies and to strengthen the commitments made by institutions concerning the most vulnerable areas and groups of individuals. The economic crisis weakens the educational and healthcare systems to the same extent that it weakens prevention and promotion measures relating to sexual health [9].

Finally, the representation of sex workers among the study population must also be considered. A previous study conducted in female sex workers in Spain shows that the prevalence of self-reported STIs experienced a significant increase between 2005 and 2011 (from 14% to 20.6%), pointing to inconsistent condom use as a factor worth considering [28]. In our study, a higher proportion of the immigrant population are sex workers compared to the native population, which may also explain the higher prevalence of STIs. However, further studies are needed to corroborate this association.

### Limitations

Caution must be exercised when interpreting the data obtained and generalising them to the immigrant population as a whole due to certain limitations of this study. For example, it should be taken into consideration that this study has been carried out in a specific geographical area. As a result, this study has a low degree of external validity. However, the close relationship that exists between population, culture, healthcare systems, and the use of healthcare services (such as STI-related services) justifies the need to perform field analyses such as this one. Additionally, given that this study has been conducted in a specific healthcare district, it provides varied and accurate local data on the composition of the population attached to this area, data that are not usually available from demographical sources.

The non-Spanish population analysed in this study is over-represented in comparison with the non-Spanish population officially resident in the province of Granada, which may indicate the large number of illegal immigrants that characterises international migration in Southern Europe. In Spain, official sources have estimated that, only during the period analysed, 195,458 undocumented individuals had arrived in Spain [29], with Granada being one of the most affected provinces [30].

Furthermore, despite having analysed a long period of time, the cross-sectional nature of this study does not allow causal associations to be established. As a result, the findings shown in this respect must be regarded as hypotheses to be tested with other more complex designs that facilitate the establishment of stronger causal relationships. There is an inconclusive result suggesting the need to further investigate the results for more conclusive outcomes in future studies.

## 5. Conclusions

During the period 2000–2014, the risk of being infected with an STI is greater in the non-Spanish population throughout the entire period analysed. There was also a gradual decrease in the rate of STI diagnoses in the immigrant population from 2009 to the lowest level of the time series in 2014, which led to a lower risk of being diagnosed with an STI during the same period. The difference in risk observed between the two populations is less marked during the crisis period (2008–2014) compared to the non-crisis period (2000–2007). This may be attributed to an underreporting of diagnoses in the immigrant population during the crisis period.

Drawing on the epidemiological and social context, the findings of this study show a population profile (of the non-Spanish population), which is more vulnerable to STIs. It should be noted that, for example, the lack of STI diagnosis and treatment in illegal immigrants hinders STI control. This, in turn, may increase STI transmission likelihood and could result in a deterioration in the health of the affected group and, potentially, of the general population [8]. Public health policies must improve the control and treatment of existing cases, allocate more resources for the detection of unreported cases, and put in place more effective preventative measures at a lower cost.

## Figures and Tables

**Table 1:** Sample description of people diagnosed with a sexually transmitted infection (STI) by nationality (Spanish or non-Spanish) (*n* = 441).

	Non-Spanish Nationals (*n* = 112; 25.39%)	Spanish Nationals (*n* = 329; 74.6%)
	*n* (%)	*n* (%)
Sex (*n* = 441)		
Male	28 (25.0)	201 (61.1)
Female	84 (75.0)	128 (38.9)
Occupation (*n* = 419)		
Sex worker/Former sex worker	64 (59.8)	8 (2.6)
Other	43 (40.2)	304 (97.4)
Employment status (*n* = 400)		
Employed	72 (73.5)	131 (43.4)
Unemployed	26 (26.5)	171 (56.6)
Level of education (*n* = 416)		
No education/Primary education	32 (32.0)	45 (14.2)
Secondary education	39 (39.0)	56 (17.7)
Vocational training/Training module	6 (6.0)	43 (13.6)
Higher education	23 (23.0)	172 (54.4)
Has regular partner (*n* = 417)		
Yes	69 (65.7)	212 (67.9)
No	36 (34.3)	100 (32.1)
Reason for visit (*n* = 441)		
Symptoms	34 (30.4)	243 (73.9)
Other	3 (2.7)	9 (2.7)
HIV	75 (67.0)	77 (23.4)
Previous treatment (*n* = 326)		
Yes	21 (27.6)	85 (34.0)
No	55 (72.4)	165 (66.0)
Sexual orientation identity (*n* = 428)		
Heterosexual	103 (94.5)	248 (77.7)
Bisexual	3 (2.8)	14 (4.4)
Homosexual	3 (2.8)	57 (17.9)
Regular partner has symptoms (*n* = 163)		
Yes	14 (34.1)	53 (43.4)
No	27 (65.9)	69 (56.6)
Drug use (*n* = 194)		
Yes	16 (34.0)	63 (42.9)
No	31 (66.0)	84 (57.1)
Previous STIs (*n* = 356)		
Yes	24 (27.0)	65 (24.3)
No	65 (73.0)	202 (75.7)
	**Mean (SD)**	**Mean (SD)**
Age (*n* = 441)	27.36 (6.08)	28.22 (8.76)
Subsequent visits (*n* = 439)	1.45 (1.24)	1.25 (1.46)
New episodes (*n* = 440)	1.06 (1.52)	0.69 (1.12)
Period of time since last sexual contact without a condom (*n* = 259)	2.36 (0.87)	2.40 (0.80)
Number of partners in the last month (*n* = 441)	3.48 (1.88)	1.34 (0.81)
Number of partners in the last year (*n* = 419)	5.03 (2.43)	2.39 (1.42)
Lifelong sexual history (*n* = 99)	2.41 (0.85)	1.84 (0.85)
Age of first sexual intercourse (*n* = 233)	17.13 (2.46)	17.62 (3.33)

SD: Standard Deviation; HIV: Human Immunodeficiency Virus. Period of time since last sexual contact without a condom: 1 = never, 2 = less than one month, 3 = one to six months, 4 = six to 12 months, 5 = more than 12 months; Number of partners in the last month: 1 = 0–1, 2 = 2, 3 = 3–5, 4 = more than 5; Number of partners in the last year: 1 = 0–1, 2 = 2, 3 = 3–5, 4 = 6–10, 5 = 11–20, 6 = more than 20; Lifelong sexual history: 1 = 0–10, 2 = 10–20, 3 = more than 20.

**Table 2:** Distribution of STIs in non-Spanish and Spanish populations (*n* = 378). Granada, Spain, 2000–2014.

	Non-Spanish Nationals (*n =* 112)	Spanish Nationals (*n* = 329)
	*n (*%)	*n (*%)
HPV (*n* = 208)	41 (36.6)	167 (50.8)
Candidiasis (*n* = 66)	29 (25.9)	37 (11.2)
Molluscum contagiosum (n = 38)	3 (2.7)	35 (10.6)
Gardnerella (*n* = 31)	18 (16.1)	13 (4.0)
Syphilis (*n* = 26)	6 (5.4)	20 (6.1)
Gonococcal infection (*n* = 25)	3 (2.7)	22 (6.7)
Herpes simplex virus (*n* = 22)	4 (3.6)	18 (5.5)
HIV (*n* = 15)	4 (3.6)	11 (3.3)
HBV (*n* = 4)	3 (2.7)	1 (0.3)
Other (*n* = 6)	1 (0.8)	5 (1.5)

HPV: Human Papilloma Virus. HIV: Human Immunodeficiency Virus. HBV: Hepatitis B Virus.

**Table 3:** Annual odds ratios for STIs in non-native populations versus native populations aged between 15 and 64 years old. Granada, Spain, 2000–2014.

Year	OR	95% CI
2000	22.51	13.18–64.32
2001	4.05	1.72–21.62
2002	9.37	5.54–26.26
2003	10.31	6.25–27.53
2004	17.54	10.87–44.77
2005	8.87	6.19–17.97
2006	21.01	14.44–43.83
2007	8.36	6.12–15.38
2008	3.69	2.59–7.41
2009	5.68	4.02–11.24
2010	3.39	2.29–7.35
2011	3.03	2.05–6.52
2012	1.80	1.08–4.94
2013	1.76	1.11–4.37
2014	0.37	0.16–1.91
2000–2014	5.33	4.78–6.60
2000–2007	12.02	10.33–16.17
2008–2014	2.73	2.32–3.73

OR: odds ratio. STIs: sexually transmitted infections. CI: confidence interval.
